# Test-retest reliability of diffusion kurtosis imaging metrics in the healthy adult brain

**DOI:** 10.1016/j.ynirp.2022.100098

**Published:** 2022-05-15

**Authors:** Liu-Yang Wu, Yao Xu, Lan-Lan Chen, Wen-Rui Yang, Yan Li, Song-An Shang, Xian-Fu Luo, Wei Xia, Jing Xia, Hong-Ying Zhang

**Affiliations:** a Department of Radiology, Northern Jiangsu People's Hospital, Yangzhou University, Yangzhou, 225000, Jiangsu Province, China; b Department of Radiology, Wuxi No.2 People's Hospital, Nanjing Medical University, Wuxi, 214000, Jiangsu Province, China; c Department of Neurology, Northern Jiangsu People's Hospital, Yangzhou University, Yangzhou, 225000, Jiangsu Province, China; d Internal Auditing Office, Northern Jiangsu People's Hospital, Yangzhou University, Yangzhou, 225000, Jiangsu Province, China

**Keywords:** Test-retest reliability, Diffusion kurtosis imaging, Gray matter, White matter, MRI

## Abstract

**Purpose:**

To evaluate the reliability of magnetic resonance imaging (MRI) diffusion kurtosis imaging (DKI) derived metrics from the human brain as well as the effect of spatial smoothing preprocess on measurement reliability through test-retest measurements.

**Materials and methods:**

A total of 23 healthy volunteers who underwent MRI DKI scanning during two sessions performed at an interval of two weeks were enrolled in the study. For the eight DKI-derived metrics, intraclass correlation coefficient (ICC) and coefficient of variation (CV) were used to assess regional and global reliability and reproducibility of based gray matter (GM) atlas in the automated anatomical labeling (AAL) and white matter (WM) atlas in JHU-White Matter-Labels. Additional comparisons were made between the reliabilities of images with and without smoothing preprocessing.

**Results:**

The average ICCs of the eight DKI metrics varied from 0.45 to 0.91, CVs ranged from 1.48% to 4.60% in global GM and WM regions, and the reliability degree ranged from moderate to almost perfect, with regional variations. Kurtosis-related metrics presented inferior reliability to the tensor-related metrics. Among the eight DKI metrics, RD had the best reliability, while KFA ranked the last. The reliability of FA in WM was superior to that in GM; however, FA did not result in a good index for GM reliability assessment. Although the DKI reliability of GM regions varied, the default mode and visual areas consistently demonstrated the highest ICC reliability for a series of kurtosis-related metrics. Additionally, smoothing preprocessing increased reliability for a few metrics only in WM compared to preprocessing without smoothing (*P* < 0.05, FDR corrected).

**Conclusion:**

The DKI metrics can be reliably used as biomarkers for diffusion property measurements. The kurtosis-related metrics presented lower reliability compared to the tensor-related metrics from DKI data. Default mode-related areas exhibited higher reliability than other brain cortices in terms of implicated microstructural DKI property. Additionally, an appropriate smoothing preprocessing could improve the reliability of a few DKI metrics only in WM regions.

## Highlights

• The reliability of the DKI metrics ranged from moderate to almost perfect, among which RD presented the best reliability.
• The tensor-related metrics presented better reliability than the kurtosis-related metrics.
• The default mode network regions seemed to present the highest reliability among the cerebral cortices for the DKI metrics.
• Appropriate spatial smoothing post-processing could improve reliability in WM but not in GM regions.

## 1 Introduction

Magnetic resonance imaging (MRI) quantification is important for the diagnosis and differential diagnosis of disorders and monitoring therapeutic effects in the human brain (Abdel Razek et al., 2019; Bergamino et al., 2020; Chenevert et al., 2000). For example, the water diffusion features presented by apparent diffusion coefficient (ADC) values can be monitored in brain glioma after non-operation treatment. Also, ADC can be used as a valuable candidate biomarker in evaluating tumor response to cytotoxic treatment (Chenevert et al., 2000). Therefore, to accurately quantify microstructural changes in cerebral disease progression, the MRI acquired metrics must be reliable and stable. In other words, before utilizing these metrics as reliable biomarkers, it is necessary to fully investigate whether they have adequate test-retest reliability. Thus far, numerous studies have investigated the test-retest reliability of structural high-resolution T1 images, diffusion tensor imaging (DTI), and BOLD functional MRI, revealing that these MRI imaging modalities had varying degrees of reliability (Merisaari et al., 2019; Rezende et al., 2019; Yang et al., 2021).

Over recent years, the development of the diffusion imaging-related technical modes has rapidly progressed, and these modalities have been widely used for quantitative evaluation of the characteristics of diffusion motion of water molecules in the human brain. Among these modes, DTI can be used to quantitatively detect the structural integrity of white matter (WM) based on the assumption that the diffusion motion of water molecules conforms to the Gaussian distribution (Ciccarelli et al., 2008; Mori and Zhang, 2006). However, the following limitations associated with DTI have been identified: first, the complexity of brain microstructure (such as cell membrane, organelles) hinders and limits the diffusion of water molecules on different length scales, resulting in non-Gaussian diffusion of water molecules (Tan, et al, 2019); second, the simple diffusion tensor model with a 2nd-order 3D diffusivity tensor cannot effectively quantify the relatively isotropic tissue structure such as gray matter (GM) (Jensen et al., 2005; Marrale, et al, 2016); third, even in WM, the DTI model fails to describe and resolve the full directional information of the diffusion process in complex or crossing fibers since the tensor has only the maximum value in one direction, i.e., the main eigenvalue of the diffusion tensor (Lazar, et al, 2008; Tuch, et al, 2002). To address the issues, diffusion kurtosis imaging (DKI), a promising extension of DTI, has been proposed to quantitatively measure non-Gaussian diffusion effect by introducing more b values in diffusion acquisition (Jensen and Helpern, 2010; Jensen et al., 2005). By using DKI data, besides the diffusion tensor-related metrics of axial diffusivity (AD), radial diffusivity (RD), fractional anisotropy (FA) and mean diffusivity (MD), it is also possible to obtain the kurtosis-related metrics of axial kurtosis (AK), radial kurtosis (RK), mean kurtosis (MK) and kurtosis fractional anisotropy (KFA). According to the literature, DKI-derived metrics can offer more comprehensive and sensitive detections of tissue microstructural changes compared to DTI-derived metrics. Besides, they are well suited for characterizing not only WM but also GM microstructure (Bester et al., 2015; Cheng et al., 2018; Winston, 2015; Zhao et al., 2019). Yet, the increasing use of DKI for human brain measurement has also resulted in ambiguous and inconsistent results reported by different studies. For example, Wang et al. (2011) found significantly increased MK and FA in the substantia nigra (SN) of patients with Parkinson's disease (PD). In contrast, a different study reported increased MK and decreased FA in SN of PD patients (Zhang et al., 2015). Therefore, it is crucial to investigate the reliability of a series of DKI metrics beforehand, especially taking into account the complexity of the human brain.

The reliability of MRI measurements does not only depend on the factors that are easily controlled, such as MRI scanning and post-processing methods, but is also related to uncontrollable factors, such as physiological noise, head positioning, and motion effect. These variabilities should be taken into account when interpreting MRI results. Nevertheless, as standard scan protocol and post-processing scheme are still not available, reliable estimation of DKI measurements is quite challenging. Recently, a few studies have explored the factors affecting DKI reliability by manipulating MRI scanning schemes, including the number of gradient sampling directions, b-values (Chiang et al., 2019; Chou et al., 2017; Yokosawa et al., 2016), and different field strengths of scanners (Shaw et al., 2017). For instance, in their study, Chiang et al. used a naive rat model, finding that compared to the scheme with six b-values and fifteen gradient directions, the scheme with three b-values and thirty gradient directions exhibited higher precision and reliability of DKI-derived metrics, especially for FA and MK (Chiang et al., 2019). On the other hand, the previous reliability tests were found to have various limitations. For example, only a few brain regions-of-interest (ROIs) (Chou et al., 2017) or lesions with considerable heterogeneity were tested (Shahim et al., 2017), and some studies only measured minor DKI metrics (Chiang et al., 2019; Yokosawa et al., 2016). Another limitation in some previous studies was the length of interval time between sessions that were overlooked (Chiang et al., 2019; Chou et al., 2017; Yokosawa et al., 2016). To date, there is little information about the systematic reliability test of DKI measurement across the whole brain in consideration of the heterogeneous brain cytoarchitecture.

Additionally, post-processing schemes can also impact the reliability of DKI measurements, including brain extraction, distortion correction, noise and motion correction, spatial smoothing, diffusion metric estimations, and so on. Among these schemes, spatial smoothing is typically used to denoise the image and improve the signal-to-noise ratio (SNR), which is a process often employed in image analysis. A previous study investigated the effect of spatial smoothing during DTI test-retest measurement (Madhyastha et al., 2014), indicating that spatial smoothing preprocess could improve the reliability; for their DTI data with voxel-size of 2.4 × 2.4 × 2.4 mm, slight smoothing with a 4 mm FWHM kernel could reduce the coefficients of variation (CV). However, it remained unclear whether the spatial smoothing preprocess had any impact on the reliability of DKI metrics.

Therefore, the aim of the present study was to perform comprehensive reliability assessments of the eight quantitative DKI metrics based on functional and structural brain sub-regions through test-retest measurements.

## 2 Materials and Methods

### 2.1 Subjects

A total of 23 healthy subjects, 13 females and 10 males, aged 25–34 years, were recruited in the study. The exclusion criteria were the following: (I) a history of neurological impairments or psychiatric disorders; (II) claustrophobia; (III) could not keep still during the examinations.

This study was approved by the Ethics Committee of Northern Jiangsu People's Hospital. All subjects were fully informed of the nature and process of the study; they all provided written informed consent before MRI scanning.

### 2.2 MRI acquisition

MRI data acquisition was performed using a 3.0T MR scanner (Discovery MR750; GE Medical Systems, Milwaukee, WI, USA) with an eight-channel phased-array head coil. All volunteers underwent two identical MRI examinations with an interval of two weeks. The conventional MRI protocol, including an axial T2-weighted, an axial T1-weighted, and an axial diffusion-weighted imaging (DWI) sequence, was first performed to exclude the presence of any lesions, after which DKI images were acquired using a spin-echo single-shot echo-planar sequence with following parameters: TR = 8000 ms, TE = 92.9 ms, matrix = 100 × 100, FOV = 250 × 250 mm^2^, NEX = 1, slice thickness = 2.5 mm, 2 b values (b = 1250 and 2500 s/mm^2^) and 30 directions at each b value. Additionally, three non-diffusion-weighted images at b = 0 s/mm^2^ were also acquired. The acquisition time was 8 min and 32 s. Moreover, the high resolution T1-weighted 3D brain volume (3D-BRAVO) images based on fast spoiled gradient echo, providing reference anatomical structure covering the whole brain, were acquired with the following parameters: TR = 8.16 ms, TE = 3.18 ms, FOV = 250 × 250 mm, matrix = 256 × 256, slice thickness = 1 mm, 180 sagittal slices. The scan time was 4 min and 2 s.

### 2.3 Image processing and analysis

The DKI raw data were pre-processed by FMRIB Software Library (FSL) version 5.0 and subsequently calculated by Diffusional Kurtosis Estimator (DKE, Version 2.6.0, https://www.nitrc.org/projects/dke) to obtain the eight metrics. SPM12 (http://www.fil.ion.ucl.ac.uk/spm) was then employed for normalization and spatial smoothing.

Data pre-processing included the removal of non-brain tissue and correction of movement and eddy current distortion. Next, kurtosis and diffusion tensor were fitted on a voxel-by-voxel basis using a constrained linear weighted algorithm with DKE software, and eight DKI-derived metrics were computed accordingly. For all resultant DKI parametric maps, we performed an affine co-registration to the skull-stripped T1 weighted 3D anatomic images.

Subsequently, T1 weighted 3D images were segmented into three components of gray matter (GM), WM, and cerebrospinal fluid, after which they were normalized into a Montreal Neurological Institute (MNI) space using a high-dimensional Diffeomorphic Anatomical Registration Through Exponentiated Lie Algebra (DARTEL) algorithm. Next, after co-registration, all DKI parametric maps were normalized to the MNI space using the deformation matrix created in the registration step. A resolution of 2.5 × 2.5 × 2.5 mm was finally used as voxel size. After data normalization, slight smoothing was performed with a 4 mm FWHM kernel size for improving image quality. Meanwhile, the smoothing kernel size was selected by referring to the previous studies (Alahmadi, 2021; Madhyastha et al., 2014; Vollmar et al., 2010) so as to control the potential false-positive ratios induced by larger kernel sizes. Images with and without smoothing were used to assess the effect of spatial smoothing on DKI reliability measurements. Then, each parametric map was fused with GM and WM ROIs, which were defined in the templates of automated anatomical labeling (AAL) and JHU-White Matter-Labels, respectively (Mori et al., 2008; Tzourio-Mazoyer et al., 2002). The corresponding region-specific parametric values were then extracted. There are 48 WM ROIs in the JHU-White Matter-Labels, including 6 unilateral structures, and 90 GM ROIs from AAL covering the bilateral cerebral cortex (excluding the cerebellum). The DKI metrics values were averaged for the bilateral symmetric ROIs due to the similar values across the symmetric structures. Therefore, the DKI metrics values from a total of 45 GM and 27 WM ROIs of each individual were used for further reliability analysis.

### 2.4 Statistical analysis

We chose intraclass the correlation coefficient (ICC) and CV as reliability indexes to estimate the degree of agreement and variability between repeated measurements. As a common measure of reliability, the ICC can be regarded as a ratio of between-subject variance divided by total variance, which can be partitioned into within- and between-subject variance. High ICC values require low measurement error within-subject, as well as high variability between-subject. Similar to previous studies (Somandepalli et al., 2015; Zuo et al., 2013), a linear mixed effects (LME) model was employed to model both within- and between-individual variability to further compute ICC as follows (Zuo and Xing, 2014):

(1) $$\Phi_{\mathrm{ij}}=\lambda_{0j}+e_{\mathrm{ij}},\cdot \lambda_{0j}=\mu_{00}+p_{0j}$$

(2) $$ICC\left(Ф\right)\;=\;\frac{\sigma_{p}^{2}}{\sigma_{p}^{2}\;+\;\sigma_{e}^{2}}$$

where *Ф*_*ij*_ denotes the measurement value of any of the eight metrics for each ROI from the jth participant's ith measurement (for i = 1, 2 and j = 1,⋯,23). The *μ*_00_ is a fixed parameter (the group mean), and *p*_*0j*_ and *e*_*ij*_, which are random effects with variances $\sigma_{p}^{2}$ and $\sigma_{e}^{2}$, denote the participant effect and measurement error, respectively.

As another commonly descriptive statistic, CV, which relates the within-subject standard deviation (SD) of the measurements to its mean, is calculated as:

(3) $$CV\;=\;\frac{SD}{Mean}*100\%$$

According to a previous report (Landis and Koch, 1977), reliability is considered slight if ICC≤0.2, fair if 0.2–0.4, moderate if 0.4–0.6, substantial if 0.6–0.8 and almost perfect with 0.8 < ICC <1.0. In the present study, measurements with ICC≥0.60 and CV ≤ 10% were considered acceptable standards (Venkatraman et al., 2015). In addition, the Kolmogorov–Smirnov tests were conducted to examine the normal distribution of variables. Next, paired *t*-tests with false discovery rate (FDR) correction between the images with and without smoothing were performed to evaluate the impact of spatial smoothing on the reliability of DKI measurements. All statistical analyses were performed using R version 3.6.1 and SPSS version 16.0. A threshold of *P* < 0.05 represented statistically significant differences.

### 2.5 Head motion and SNR measurement

To control potential impact of head motion on the reliability measurement, six translation and rotation parameters of head motion of the DKI data volumes were calculated though SPM12 and assessed for quality control. Due to high b values used during DKI data acquisition, the SNR of the DKI data could be pretty low and impact on the reliability measurement. We calculated and compared the SNR between the two b values of 1250 and 2500 in the DKI raw data. The SNR calculation method referred to the previous work (Shaw et al., 2017).

## 3 Results

### 3.1 Assessments of head motion and image SNR

The comparisons of each head motion parameter between sessions of each individual showed no significant differences by two-example *t*-test (*P* values 0.29–0.76). For all the subjects, the SNR values at b_1250_ and b_2500_ images were 19.84 ± 4.77 and 11.26 ± 2.65, respectively. There were significant differences in comparison between the SNRs of b_1250_ and b_2500_ with paired two-sample test (*P* < 0.001). The SNR at the b_2500_ image was about half of that at the b_1250_ image.

### 3.2 Test-retest reliability of DKI measurements

**Fig. 1:**
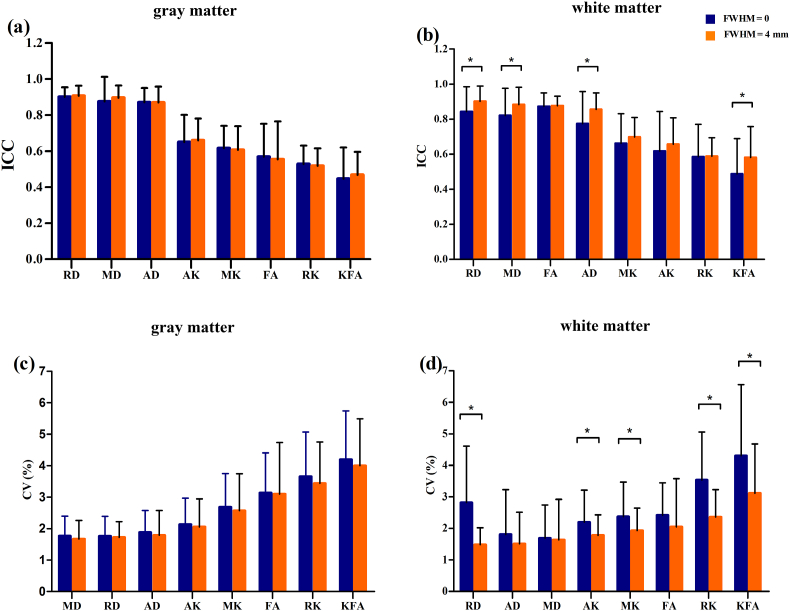
The ranked reliability degree expressed by the ICC and CV values (mean ± standard deviation) of eight DKI metrics in gray matter and white matter. The tensor-related metrics mostly ranked ahead of the kurtosis-related metrics, with RD having the highest and KFA having the lowest reliabilities. Paired *t*-test comparisons between unsmoothed and smoothed images were statistically significant in white matter for ICC in RD, MD, AD, KFA, and for CV in RD, AK, MK, RK, and KFA (**b, d**), whereas no significant differences were found in gray matter for smoothing effect **(a, c)**. *: *P* < 0.05, corrected by FDR. Error bars represent the group standard deviation of the ICC and CV values. ICC: intraclass correlation coefficient, CV: coefficient of variation.

**Fig. 2:**
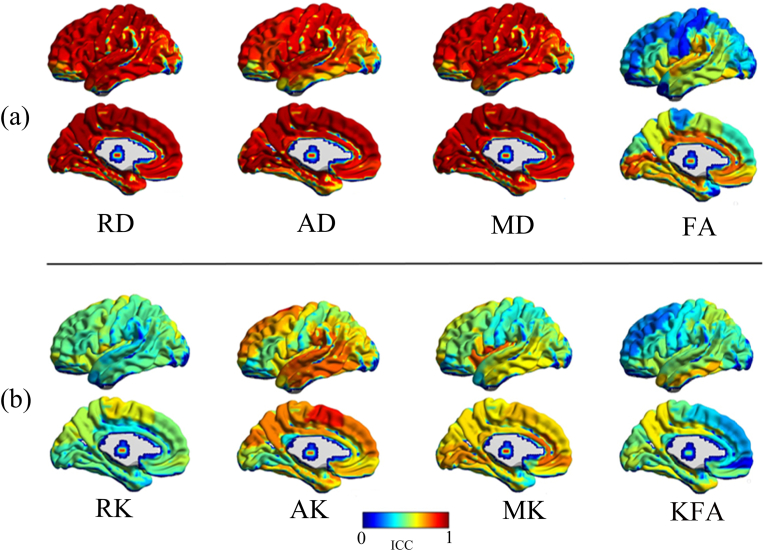
The reliability degree map represented by AAL gray matter ROI-wise ICC sizes. **(a)** Tensor-derived metrics; **(b)** kurtosis-derived metrics. The color bar means the ICC value size. Noted from the mapping, the kurtosis-related metrics generally presented inferior reliability to the diffusion tensor-related metrics, and default mode areas, as well as the visual cortex, showed high agreement with the highest reliability for FA, RK, AK, MK, RKA metrics. AAL: automated anatomical labeling. (For interpretation of the references to color in this figure legend, the reader is referred to the Web version of this article.)

For GM and WM ROIs, the average ICCs of the eight metrics varied from 0.45 to 0.91, while CVs ranged from 1.48% to 4.60%. The reliability degree of the eight metrics was ranked, and it ranged from moderate to almost perfect. RD was found to have the highest reliability, by and large, followed by MD, AD, AK, MK, FA, RK, and KFA (Fig. 1). Moreover, the kurtosis-related metrics revealed inferior reliability to the tensor-related metrics (Figs. 1 and 2). The ICC and CV values for eight DKI-derived metrics obtained from 45 GM and 27 WM ROIs are shown in Table 1, Table 2, Table 3, Table 4, respectively.

**Table 1:** The ICC value of for eight DKI metrics in ROIs each of AAL.

ROI	AD	RD	MD	FA	AK	RK	MK	KFA
Precentral gyrus	0.88	0.94	0.93	0.25	0.65	0.54	0.48	0.38
Superior frontal gyrus, dorsolateral	0.89	0.93	0.92	0.31	0.75	0.48	0.58	0.28
Superior frontal gyrus, orbital part	0.85	0.79	0.83	0.42	0.60	0.59	0.67	0.38
Middle frontal gyrus	0.85	0.91	0.89	0.21	0.70	0.48	0.52	0.23
Middle frontal gyrus, orbital part	0.56	0.72	0.66	0.15	0.36	0.46	0.32	0.45
Inferior frontal gyrus, opercular part	0.91	0.95	0.94	0.56	0.65	0.50	0.68	0.50
Inferior frontal gyrus, triangular part	0.82	0.88	0.86	0.43	0.49	0.44	0.50	0.42
Inferior frontal gyrus, orbital part	0.80	0.86	0.84	0.54	0.52	0.58	0.54	0.55
Rolandic operculum	0.91	0.94	0.94	0.73	0.68	0.66	0.84	0.37
Supplementary motor area	0.96	0.97	0.96	0.56	0.86	0.60	0.68	0.34
Olfactory cortex	0.93	0.89	0.91	0.72	0.69	0.47	0.48	0.48
Superior frontal gyrus, medial	0.94	0.93	0.94	0.43	0.75	0.51	0.65	0.29
Superior frontal gyrus, medial orbital	0.83	0.87	0.86	0.64	0.64	0.56	0.75	0.12
Gyrus rectus	0.90	0.93	0.92	0.75	0.69	0.47	0.63	0.51
Insula	0.95	0.92	0.93	0.66	0.65	0.56	0.77	0.55
Anterior cingulate and paracingulate gyri	0.87	0.90	0.89	0.78	0.63	0.49	0.77	0.38
Median cingulate and paracingulate gyri	0.94	0.94	0.94	0.77	0.74	0.39	0.65	0.63
Posterior cingulate gyrus	0.85	0.86	0.89	0.74	0.38	0.47	0.69	0.42
Hippocampus	0.88	0.92	0.91	0.77	0.68	0.53	0.69	0.65
Parahippocampal gyrus	0.96	0.95	0.96	0.73	0.69	0.50	0.64	0.51
Amygdala	0.94	0.91	0.92	0.74	0.54	0.44	0.41	0.28
Calcarine fissure and surrounding cortex	0.95	0.95	0.95	0.81	0.56	0.44	0.71	0.57
Cuneus	0.90	0.93	0.92	0.49	0.76	0.55	0.65	0.50
Lingual gyrus	0.96	0.94	0.95	0.66	0.47	0.49	0.73	0.49
Superior occipital gyrus	0.78	0.91	0.88	0.29	0.66	0.62	0.61	0.43
Middle occipital gyrus	0.72	0.84	0.81	0.36	0.67	0.51	0.54	0.47
Inferior occipital gyrus	0.82	0.91	0.89	0.65	0.75	0.51	0.62	0.46
Fusiform gyrus	0.78	0.89	0.85	0.74	0.72	0.50	0.66	0.64
Postcentral gyrus	0.90	0.96	0.94	0.15	0.49	0.46	0.40	0.39
Superior parietal gyrus	0.91	0.98	0.96	0.25	0.54	0.47	0.41	0.55
Inferior parietal, but supramarginal and angular gyri	0.91	0.94	0.93	0.49	0.67	0.46	0.45	0.56
Supramarginal gyrus	0.93	0.92	0.92	0.70	0.78	0.39	0.49	0.46
Angular gyrus	0.83	0.90	0.88	0.52	0.67	0.50	0.51	0.43
Precuneus	0.96	0.97	0.97	0.59	0.75	0.55	0.65	0.64
Paracentral lobule	0.93	0.96	0.95	0.26	0.72	0.65	0.62	0.48
Caudate nucleus	0.97	0.98	0.98	0.81	0.92	0.82	0.91	0.70
Lenticular nucleus, putamen	0.88	0.85	0.84	0.79	0.61	0.62	0.53	0.47
Lenticular nucleus, pallidum	0.85	0.73	0.67	0.75	0.38	0.62	0.61	0.22
Thalamus	0.88	0.91	0.90	0.79	0.79	0.80	0.86	0.54
Heschl gyrus	0.94	0.93	0.94	0.67	0.68	0.54	0.75	0.47
Superior temporal gyrus	0.90	0.91	0.91	0.73	0.80	0.34	0.53	0.50
Temporal pole: superior temporal gyrus	0.90	0.95	0.94	0.37	0.76	0.49	0.48	0.48
Middle temporal gyrus	0.82	0.89	0.87	0.56	0.77	0.44	0.62	0.52
Temporal pole: middle temporal gyrus	0.63	0.84	0.76	0.05	0.63	0.30	0.35	0.67
Inferior temporal gyrus	0.67	0.90	0.83	0.56	0.78	0.48	0.63	0.70
Mean ± standard deviation	0.87 ± 0.09	0.91 ± 0.06	0.90 ± 0.07	0.55 ± 0.21	0.66 ± 0.12	0.52 ± 0.10	0.61 ± 0.13	0.47 ± 0.13

*ICC* intraclass correlation coefficient, *DKI* diffusion kurtosis imaging, *ROI* region of interest, *AD* axial diffusivity, *RD* radial diffusivity, *MD* mean diffusivity, *FA* fractional anisotropy, *AK* axial kurtosis, *RK* radial kurtosis, *MK* mean kurtosis, *KFA* kurtosis fractional anisotropy.

**Table 2:** The ICC value for eight DKI metrics in each white matter ROIs.

ROI	AD	RD	MD	FA	AK	RK	MK	KFA
Middle cerebellar peduncle	0.77	0.84	0.82	0.90	0.71	0.59	0.77	0.45
Pontine crossing tract	0.77	0.89	0.78	0.82	0.62	0.83	0.67	0.34
Genu of corpus callosum	0.95	0.93	0.94	0.89	0.74	0.70	0.73	0.76
Body of corpus callosum	0.81	0.94	0.90	0.87	0.63	0.53	0.60	0.52
Splenium of corpus callosum	0.87	0.96	0.93	0.92	0.41	0.40	0.58	0.60
Fornix (column and body of fornix)	0.95	0.97	0.97	0.88	0.69	0.76	0.80	0.61
Corticospinal tract	0.79	0.82	0.78	0.83	0.82	0.71	0.77	0.74
Medial lemniscus	0.57	0.54	0.54	0.85	0.74	0.57	0.59	0.23
Inferior cerebellar peduncle	0.86	0.94	0.92	0.84	0.61	0.54	0.65	0.61
Superior cerebellar peduncle	0.92	0.96	0.96	0.73	0.78	0.50	0.76	0.58
Cerebral peduncle	0.86	0.87	0.87	0.86	0.57	0.42	0.63	0.29
Anterior limb of internal capsule	0.90	0.84	0.88	0.88	0.51	0.59	0.61	0.45
Posterior limb of internal capsule	0.75	0.86	0.78	0.87	0.53	0.74	0.57	0.79
Retrolenticular part of internal capsule	0.92	0.91	0.92	0.84	0.67	0.51	0.66	0.60
Anterior corona radiata	0.95	0.95	0.95	0.88	0.87	0.64	0.88	0.82
Superior corona radiata	0.92	0.95	0.94	0.92	0.74	0.69	0.79	0.67
Posterior corona radiata	0.92	0.98	0.97	0.93	0.89	0.70	0.89	0.86
Posterior thalamic radiation	0.91	0.98	0.96	0.93	0.81	0.61	0.81	0.85
Sagittal stratum	0.93	0.98	0.98	0.92	0.60	0.51	0.79	0.69
External capsule	0.88	0.92	0.90	0.93	0.44	0.52	0.61	0.47
Cingulum (cingulate gyrus)	0.63	0.78	0.68	0.86	0.55	0.53	0.36	0.52
Cingulum (hippocampus)	0.95	0.98	0.97	0.96	0.60	0.67	0.74	0.55
Fornix (stria terminalis)	0.85	0.90	0.88	0.91	0.35	0.56	0.74	0.65
Superior longitudinal fasciculus	0.81	0.91	0.91	0.85	0.81	0.44	0.61	0.79
Superior fronto-occipital fasciculus	0.94	0.90	0.93	0.73	0.81	0.49	0.71	0.27
Uncinate fasciculus	0.82	0.92	0.89	0.94	0.38	0.53	0.73	0.41
Tapetum	0.91	0.92	0.92	0.92	0.85	0.60	0.80	0.60
Mean ± standard deviation	0.86 ± 0.10	0.90 ± 0.09	0.88 ± 0.10	0.88 ± 0.06	0.66 ± 0.15	0.59 ± 0.11	0.70 ± 0.11	0.58 ± 0.18

*ICC* intraclass correlation coefficient, *DKI* diffusion kurtosis imaging, *ROI* region of interest, *AD* axial diffusivity, *RD* radial diffusivity, *MD* mean diffusivity, *FA* fractional anisotropy, *AK* axial kurtosis, *RK* radial kurtosis, *MK* mean kurtosis, *KFA* kurtosis fractional anisotropy.

**Table 3:** The CV (%) value for eight DKI metrics of each of gray matter ROIs in AAL.

ROI	AD	RD	MD	FA	AK	RK	MK	KFA
Precentral gyrus	2.53	2.00	2.06	5.00	2.57	4.71	3.92	3.97
Superior frontal gyrus, dorsolateral	2.42	2.05	2.07	4.03	2.30	5.14	3.57	5.67
Superior frontal gyrus, orbital part	1.15	1.56	1.29	2.85	1.98	3.06	2.12	4.90
Middle frontal gyrus	2.60	2.02	2.18	5.00	2.30	5.16	3.71	5.92
Middle frontal gyrus, orbital part	3.04	2.59	2.70	5.06	3.40	4.22	3.95	6.65
Inferior frontal gyrus, opercular part	1.62	1.33	1.36	2.58	1.36	2.33	1.53	3.53
Inferior frontal gyrus, triangular part	2.45	2.19	2.24	3.43	2.10	3.42	2.65	4.28
Inferior frontal gyrus, orbital part	2.32	1.92	2.01	2.47	2.08	2.01	2.28	3.13
Rolandic operculum	0.93	1.07	0.88	2.22	1.41	2.17	1.49	3.49
Supplementary motor area	1.69	1.56	1.52	2.31	1.65	3.59	2.67	3.69
Olfactory cortex	1.28	2.44	1.89	2.05	2.17	2.91	2.39	2.85
Superior frontal gyrus, medial	1.79	1.88	1.79	3.39	2.03	4.74	2.68	5.96
Superior frontal gyrus, medial orbital	1.37	1.46	1.37	2.62	2.25	3.49	1.92	6.97
Gyrus rectus	2.16	2.09	2.11	2.09	2.19	2.35	2.18	3.79
Insula	0.80	1.42	1.09	1.71	1.31	1.58	1.21	2.61
Anterior cingulate and paracingulate gyri	1.11	1.27	1.06	2.32	1.54	2.64	1.43	4.22
Median cingulate and paracingulate gyri	0.94	1.26	1.09	1.28	1.15	2.54	1.17	2.83
Posterior cingulate gyrus	1.06	1.51	0.94	2.28	1.76	4.79	2.04	2.78
Hippocampus	1.39	2.08	1.76	1.51	1.23	3.01	2.12	1.71
Parahippocampal gyrus	1.04	1.36	1.19	1.84	1.41	1.19	1.44	2.97
Amygdala	1.26	2.11	1.68	2.10	2.10	2.66	1.98	3.58
Calcarine fissure and surrounding cortex	0.98	1.19	1.07	1.69	1.65	3.16	1.71	3.60
Cuneus	2.33	2.08	1.99	3.23	2.14	3.71	2.70	4.12
Lingual gyrus	0.88	1.30	1.12	1.39	1.50	3.27	1.65	3.63
Superior occipital gyrus	3.13	2.15	2.46	4.97	3.32	4.95	4.08	4.86
Middle occipital gyrus	2.24	1.75	1.81	3.75	2.20	4.45	3.27	5.04
Inferior occipital gyrus	1.51	1.34	1.39	3.01	2.20	5.09	3.02	4.73
Fusiform gyrus	1.01	1.06	0.97	2.03	1.38	2.92	1.89	2.65
Postcentral gyrus	2.63	1.79	2.13	5.88	3.31	5.23	4.39	4.89
Superior parietal gyrus	3.31	1.65	2.28	9.23	5.02	6.91	6.32	9.62
Inferior parietal, but supramarginal and angular gyri	2.18	2.08	2.05	3.40	2.25	4.35	3.31	4.52
Supramarginal gyrus	1.16	1.35	1.21	1.97	1.14	2.98	1.78	4.22
Angular gyrus	2.00	1.68	1.76	3.32	1.76	3.70	2.66	4.17
Precuneus	1.41	1.15	1.22	2.49	2.00	2.58	2.05	3.16
Paracentral lobule	2.67	2.22	2.39	4.23	3.04	5.02	4.32	4.27
Caudate nucleus	1.42	1.78	1.59	1.78	1.18	2.05	1.45	1.93
Lenticular nucleus, putamen	0.67	0.75	0.60	1.09	1.11	1.72	1.96	1.32
Lenticular nucleus, pallidum	1.07	1.88	1.18	2.32	1.64	5.15	3.32	2.40
Thalamus	2.19	2.70	2.41	1.53	1.51	1.88	1.70	2.07
Heschl gyrus	1.40	1.63	1.51	2.68	1.34	1.67	1.19	4.52
Superior temporal gyrus	1.27	1.18	1.17	2.23	1.13	2.39	1.49	3.57
Temporal pole: superior temporal gyrus	2.33	1.70	1.87	5.63	2.69	2.89	3.19	4.05
Middle temporal gyrus	1.60	1.23	1.32	2.76	1.46	2.27	1.63	3.45
Temporal pole: middle temporal gyrus	4.40	3.38	3.82	7.43	5.31	5.92	5.83	4.95
Inferior temporal gyrus	1.67	1.08	1.32	2.92	1.81	2.71	2.47	2.74
Mean ± standard deviation	1.79 ± 0.80	1.72 ± 0.51	1.66 ± 0.60	3.09 ± 1.66	2.05 ± 0.90	3.44 ± 1.33	2.57 ± 1.18	4.0 ± 1.51

*CV* coefficient of variation, *DKI* diffusion kurtosis imaging, *ROI* region of interest, *AD* axial diffusivity, *RD* radial diffusivity, *MD* mean diffusivity, *FA* fractional anisotropy, *AK* axial kurtosis, *RK* radial kurtosis, *MK* mean kurtosis, *KFA* kurtosis fractional anisotropy.

**Table 4:** The CV (%) value for eight DKI metrics of each of white matter ROIs.

ROI	AD	RD	MD	FA	AK	RK	MK	KFA
Middle cerebellar peduncle	3.41	1.25	3.99	4.54	1.98	2.45	1.56	2.73
Pontine crossing tract	1.51	1.72	1.15	1.22	2.01	3.20	2.02	1.71
Genu of corpus callosum	1.49	2.00	2.44	3.51	1.34	2.58	2.39	3.91
Body of corpus callosum	1.99	1.63	1.55	1.77	2.04	2.40	3.08	6.58
Splenium of corpus callosum	1.65	1.12	1.49	1.59	2.00	2.19	2.19	4.02
Fornix (column and body of fornix)	1.64	2.23	1.68	1.91	2.40	4.27	2.97	4.62
Corticospinal tract	3.57	1.96	4.80	6.08	2.24	3.05	2.59	3.10
Medial lemniscus	4.70	1.68	5.57	6.53	3.41	3.73	3.55	4.27
Inferior cerebellar peduncle	1.39	1.61	1.44	1.55	2.02	3.00	2.18	3.08
Superior cerebellar peduncle	1.97	2.98	1.61	1.82	2.67	3.20	2.02	4.10
Cerebral peduncle	1.21	1.55	1.55	2.50	2.28	3.07	2.69	4.34
Anterior limb of internal capsule	0.62	1.09	0.70	1.09	1.40	1.42	1.37	2.48
Posterior limb of internal capsule	0.74	1.08	0.67	1.25	1.23	1.12	1.31	1.49
Retrolenticular part of internal capsule	1.34	0.87	1.59	1.99	1.63	1.55	1.67	2.47
Anterior corona radiata	0.53	1.13	0.60	0.90	0.87	1.21	0.97	1.02
Superior corona radiata	0.55	0.81	0.50	0.67	0.85	1.44	0.82	0.87
Posterior corona radiata	0.97	1.03	0.60	0.57	0.91	1.47	0.90	1.11
Posterior thalamic radiation	0.98	0.86	0.76	0.83	1.17	1.07	1.26	1.69
Sagittal stratum	0.72	0.96	0.66	0.87	1.11	1.93	1.32	2.14
External capsule	0.63	0.87	0.50	0.56	1.44	1.69	1.33	1.15
Cingulum (cingulate gyrus)	1.31	2.62	1.05	1.57	2.02	2.53	2.48	7.13
Cingulum (hippocampus)	1.29	1.39	1.36	1.52	1.84	3.42	1.49	2.24
Fornix (stria terminalis)	1.32	1.36	1.85	2.47	1.89	1.93	2.60	4.57
Superior longitudinal fasciculus	0.72	1.24	0.48	0.80	0.75	1.26	1.19	2.29
Superior fronto-occipital fasciculus	0.72	1.89	1.06	1.62	1.26	2.97	1.75	3.53
Uncinate fasciculus	1.16	1.78	1.21	1.74	2.79	2.56	1.67	3.96
Tapetum	2.69	1.17	3.18	3.74	2.42	3.04	2.66	3.53
Mean ± standard deviation	1.51 ± 1.01	1.48 ± 0.55	1.63 ± 1.31	2.04 ± 1.56	1.78 ± 0.66	2.36 ± 0.88	1.93 ± 0.73	3.12 ± 1.59

*CV* coefficient of variation, *DKI* diffusion kurtosis imaging, *ROI* region of interest, *AD* axial diffusivity, *RD* radial diffusivity, *MD* mean diffusivity, *FA* fractional anisotropy, *AK* axial kurtosis, *RK* radial kurtosis, *MK* mean kurtosis, *KFA* kurtosis fractional anisotropy.

In GM regions, RD, MD and AD showed almost perfect test-retest reliability in most of the ROIs with mean ICC values of 0.91, 0.90, and 0.87, respectively. AK, MK, FA, RK, and KFA revealed substantial to moderate reliability and the corresponding mean ICC values were 0.66, 0.61, 0.55, 0.52, and 0.47, respectively (Table 1). Except for FA, the tensor-derived metrics presented superior reliability to the kurtosis-derived metrics in GM (Figs. 1a and 2). Meanwhile, a few ROIs, mainly in pallidum and near the basicranial regions, had relatively low ICCs, corresponding to low reliability (Table 1). In addition, the reliability of FA in GM was lower than in WM (*P* < 0.05, mean ICC, 0.55 vs. 0.88). According to the above acceptable reliability standard (ICC>0.6), the ICCs of FA, RK, and KFA for GM did not reach the standard.

In WM regions, all tensor-related metrics (i.e., AD, RD, FA, and MD) had almost perfect reliability (mean ICCs ≥0.8 for all). As to kurtosis-related metrics, MK and AK showed substantial reliability, followed by RK and KFA with moderate reliability, with mean ICC values of 0.70, 0.66, 0.59, and 0.58, respectively (Table 2). The kurtosis-related metrics had inferior reliability to the diffusion tensor-related metrics in WM (Fig. 1b and c). Low ICCs were mainly found in some regions with small volumes, such as medial lemniscus and cingulum for AD, RD, and MD; superior cerebellar peduncle and superior fronto-occipital fasciculus for FA, and superior fronto-occipital fasciculus and medial lemniscus for KFA (Table 2). The ICCs of RK and KFA for WM did not reach the acceptable reliability standard.

For the CV index, all observed DKI metrics primarily showed low variabilities, with mean CVs ranging from 1.66% to 4.0% and 1.48% to 3.12% in GM and WM regions, respectively (Table 3, Table 4). Unsurprisingly, ICC and CV seemed to generate a similar pattern of metric reliability, with a tendency of lower ICC for higher CV. RD, AD, and MD showed the lowest variability with mean CVs≤2%, whereas KFA presented the highest CV, with AK, MK, and RK in the middle (Table 4, Fig. 1).

### 3.3 The distribution characteristics of reliability across the cerebral cortex

The whole cerebral cortex regions consistently showed general perfect reliability for RD, AD, and MD metrics; however, for FA, AK, RK, MK, and KFA, in spite of varied reliability presented by the cortex regions, higher ICC reliability was found in the default mode areas (e.g., medial parieto-frontal cortex and lateral temporal cortex) and visual cortex, compared with other brain cortexes (Fig. 2).

### 3.4 Effect of smoothing on reliability

To test the effect of spatial smoothing preprocessing on the reliability of DKI measurements, we performed the comparison of ICC and CV between smoothed and unsmoothed DKI images, respectively. Spatial smoothing led to significant differences in ICC and CV values in WM but not in GM (Fig. 1). Specifically, RD, MD, AD, and KFA showed significantly lower ICC in unsmoothed images than in smoothed images in WM regions (*P* < 0.05, corrected by FDR) (Fig. 1b). With reference to the CV index, significantly higher values were found in RD, AK, MK, RK, and KFA for unsmoothed images compared to smoothed images in WM regions (*P* < 0.05, corrected by FDR) (Fig. 1d).

## 4 Discussion

Following the increasing clinical applications of DKI, systematical clarification of the test-retest reliability of DKI-metrics is of utmost importance. In the present study, we provided a systematic reliability evaluation of a series of DKI-derived metrics covering almost the whole cerebrum as well as cerebellar white matter through test-retest measurements in two sessions. Our main findings suggested that the reliability of DKI metrics ranged from moderate to almost perfect, with tensor-related metrics generally performing better than kurtosis-related metrics. Regardless of varied reliability distributed across brain cortices, the default mode regions hold multiple diffusion kurtosis metrics complexities as well as high reliability. Only for a few metrics in WM, slight smoothing-preprocessing could promote reliability measurement.

In the present study, the diffusion tensor-related metrics of RD, MD, and AD in both GM and WM regions and FA in WM regions were found to have almost perfect reliability, which is consistent with a previous test-retest study on DTI (Albi et al., 2017). Additionally, our findings indicated that the reliability of FA in WM was superior to that in GM, which was not surprising, considering that FA is a metric reflecting anisotropy and GM is of structural isotropy relative to WM (Rulseh et al., 2013). Accordingly, FA may not be an ideal biomarker to the quantification of GM.

In the present study, the DKI metrics revealed moderate to substantial reliability. The test-retest reliability of AK, RK, and MK as well as the four tensor-related metrics within a few WM ROIs in seven subjects, have been previously measured (Shahim et al., 2017), revealing reliability that matched with ours. Contrary to the observations from this previous study, an intriguing finding in our study was that the kurtosis-related metrics generally presented inferior reliability in both GM and WM to the tensor-related metrics. To the best of our knowledge, no previous studies have reported similar findings. This result may be theoretically explained by the kurtosis-related metrics estimation being more dependent on the high b values than the diffusion tensor-related metrics. As is well known, in order to more accurately characterize the non-Gaussian diffusion of water molecules in biological tissues, DKI scanning requires at least 2 high b-values and 15 diffusion directions so as to well fit in the fourth-order model (Jensen and Helpern, 2010). In contrast, the diffusion tensor-related metrics estimation is mostly based on the data with one low b-value, where SNR is still sufficiently high, and the influence of noise on metrics estimation and reliability is relatively small (Andre et al., 2014). Strong diffusion weighting estimation with high b values, could result in rapid SNR drop, and consequently, low SNR leads to decreased reliability (Landman et al., 2007; Li et al., 2014), as also demonstrated in our results. So, these differences in scanning schemes and algorithms could lead to more reliable tensor-related metrics than kurtosis-related metrics.

Our findings indicated that the reliability degree of DKI metrics across whole cerebral cortices varied, revealing some peculiar distribution characteristics. The default mode network regions seemed to present higher reliability than the other cortex areas, which is consistent with the test-retest reliability of network hubs from resting-state functional MRI data (Liao et al., 2013). These findings support the premise that default mode regions as the hub of brain networks not only implicate a basis of the microstructure of complicated diffusion kurtosis properties but also have very good stability. The default mode network is of special interest given its pivotal role in multiple functions, such as response to intrinsic or external information, development, disease, social cognition, and similar (Rebello et al., 2018; Spetsieris et al., 2015; Yeshurun et al., 2021). At the resting state, the default mode regions presented high metabolism, blood flow and activity (Passow et al., 2015; Raichle ME et al., 2001; Zou et al., 2009). There were very solid structural underpinnings for maintaining and facilitating the functions. As observed in our findings, the default mode regions were distinct among the cortices, consistently showing excellent reliability for the DKI metrics.

Our results revealed lower reliability in the pallidum and some basicranial GM regions for some metrics, which may be partly attributed to basicranial regions vulnerable to tissue susceptibility and image artifacts (Irfanoglu et al., 2015).

The post-processing pipeline could impact test-retest reliability. Herein, we assessed the effect of spatial smoothing on the reliability of DKI measurements, finding that spatial smoothing significantly increased the ICCs of AD, RD, MD, and KFA and reduced the CVs of RD, AK, RK, MK, and KFA in WM regions. These results suggested that appropriate smoothing could enhance the reliability of DKI measurements in WM regions, partly because spatial smoothing could also reduce noise levels and improve image quality (Xing et al., 2011). Still, in contrast to WM, GM was not found to have altered reliability with the application of smoothing, which could be attributed to the relatively structural homogeneity of gray matter, increasing the tendency toward diffusion isotropy and causing insensitivity to the smoothing processing. On the contrary, WM is basically constituted by fiber bundles with multiple directions, which leads to diffusion anisotropy, thus making the voxels to be sensitive to the smoothing processing.

There are several limitations in the present study. First, our data were obtained from only a small sample of adults. Second, there is still no standard procedure for DKI acquisition, and DKI images usually have low SNR due to the application of high b values.

In conclusion, the general reliability of eight DKI metrics ranged from moderate to almost perfect and could serve as biomarkers of the brain. Importantly, tensor-related metrics had better reliability than kurtosis-related metrics. Despite the varied reliability among the brain cortices, default mode-related areas consistently presented better reliability than the other cortices in terms of implicated microstructural DKI property. In addition, an appropriate smoothing procedure could make the difference between GM and WM reliability measurements.

## Funding

This study was supported by the 10.13039/501100001809National Natural Science Foundation of China (grant No. 81471642).

## Declaration of competing interest

The authors declare that they have no known competing financial interests or personal relationships that could have appeared to influence the work reported in this paper.
