# Supplementary material for: Test-retest reliability of diffusion kurtosis imaging metrics in the healthy adult brain
Source: Neuroimage Rep. 2022 May 15;2(3):100098. doi: 10.1016/j.ynirp.2022.100098 (PMC12172791; doi:10.1016/j.ynirp.2022.100098)
Supplement: Multimedia component 1 [file mmc1.pdf]

• MedSci •

Editing By Professional Editors

CERTIFICATE OF ENGLISH EDITING

This document certifies that the manuscript entitled "Test-retest reliability of diffusion kurtosis imaging metrics in the healthy adult brain" was proofread and edited for proper English language, grammar, punctuation, spelling, and overall style by one or more of the qualified scientific editors at MedSci, all of whom are native English speakers. Neither the research content nor the authors' intentions were altered in any way during the editing process. Documents receiving this certification should be English-ready for publication; however, the author can accept or reject our suggestions and changes. To see the final MedSci edited version, please visit our verification page. If you have any questions or concerns about this document or certification, please contact us at editing@medsci.cn.

Corresponding Author:Hong-Ying Zhang

DATE:2022-04-20

SIGNATURE: MedSci

CODE:0420-8116-DD21-1E30-4973

This certificate may be verified at

https://editing.medscihealthcare.com/dist/medsci-order/#/verify
